# Person-Centered Dementia Care: Workforce and Organizational Supports in Nursing Homes

**DOI:** 10.1093/geroni/igaa057.2317

**Published:** 2020-12-16

**Authors:** Jennifer Morgan, Elisabeth Burgess, Yun-Zih Chen

**Affiliations:** Georgia State University, Atlanta, Georgia, United States

## Abstract

Person-centered care that supports choice and autonomy of residents is foundational to implementing high quality care for long-term care residents including those living with dementia. Organizational change that supports person-centered approaches requires leadership engagement and a stable, well-qualified dementia-capable workforce. This paper uses survey data from a statewide probability sample of nursing homes and their staff (N=438) to identify barriers and facilitators to person-centered care. Findings show key barriers to delivering person-centered care, including a lack of staff empowerment practices and irregular use of consistent assignment. While most organizations are implementing some person-centered resident care practices, few have moved beyond predominantly institutional practices. Staff stability and retention also remain key barriers to person-centered care. Facilitators include adoption of evidence-based and inclusive quality improvement strategies. The paper will discuss implications for state, community and organizational strategies for improving staff empowerment, integrating dementia care competencies, promoting culture change and increasing leadership engagement.

